# Endoscopic removal of fungal balls using an access sheath and bendable suction device during URS: A novel approach^☆^

**DOI:** 10.1016/j.eucr.2026.103404

**Published:** 2026-03-11

**Authors:** Mies van den Biggelaar, Manon te Dorsthorst, Xiaoye Zhu, Frank d'Ancona

**Affiliations:** Department of Urology, Radboud University Medical Center, Geert Grooteplein Zuid 10, 6525, GA, Nijmegen, the Netherlands

**Keywords:** Fungal ball, Endourology, Ureterorenoscopy, Percutaneous nephrolithotomy, Candida urosepsis

## Abstract

We report the case of a high-risk patient with candidemia and obstructive uropathy caused by renal fungal balls. Initial management with systemic antifungals and stenting was insufficient. Given the patient's dual antiplatelet therapy, we opted for flexible ureterorenoscopy utilizing a bendable ureteral access sheath with an integrated suction device. This enabled in toto removal of fungal balls and efficient clearance of a concomitant stone using laser lithotripsy. To our knowledge, this is the first report demonstrating in toto removal of renal fungal balls using suction-assisted URS in a high-risk patient, offering a minimally invasive alternative to PNL.

## 1 Introduction

Ureteroscopy (URS) is an established procedure for the management of obstructing renal and ureteral stones. However, their use in cases complicated by fungal balls remains a rare and underreported area in endourology. Fungal balls (fungal bezoars) are aggregations of fungal elements, necrotic debris, and sometimes mineralized cores, which can obstruct the urinary tract and lead to hydronephrosis, urosepsis, or even anuria.^1^^,^^2^

These infections are most commonly caused by *Candida albicans*, though non-albicans species such as *Candida glabrata* and *Candida tropicalis* are increasingly reported, particularly in immunocompromised patients 3, 4, 5. Risk factors include diabetes mellitus, prolonged catheterization, antibiotic use, and urinary tract abnormalities.^3^^,^^6^

Management typically requires a multimodal approach. Systemic antifungal therapy alone is often insufficient due to poor penetration into dense fungal masses or biofilms.^7^ Endourological interventions such as percutaneous nephrostomy (PCN), double-J stenting, and flexible URS are frequently employed to relieve obstruction and facilitate direct removal or irrigation of the fungal material.^2^^,^^6^^,^^8^

Despite these strategies, complete clearance of fungal balls remains technically demanding by using URS alone. While stone baskets have been used successfully for fungal ball retrieval,^6^ their effectiveness may be limited in cases where the fungal material is friable or extensive, potentially increasing the risk of residual fragments. This case report presents a novel endoscopic approach using ureteral access sheath with an integrated suction device during URS, which enabled in toto removal of fungal balls and efficient clearance of an associated obstructing stone. This minimal invasive technique proved particularly valuable in a high-risk patient with candidemia and dual antiplatelet therapy.

## 2 Case report

A 79-year-old woman with a history of diabetes mellitus, peripheral vascular disease, congestive heart failure, prior coronary artery bypass grafting, bioprosthetic aortic valve replacement, and ascending aorta replacement presented to the emergency department with acute suprapubic and lower abdominal pain persisting for two days. The pain was initially severe and sudden in onset, occurring at rest while in bed. It partially subsided with analgesics but recurred the following night, prompting her to seek medical attention.

**Fig. 1:**
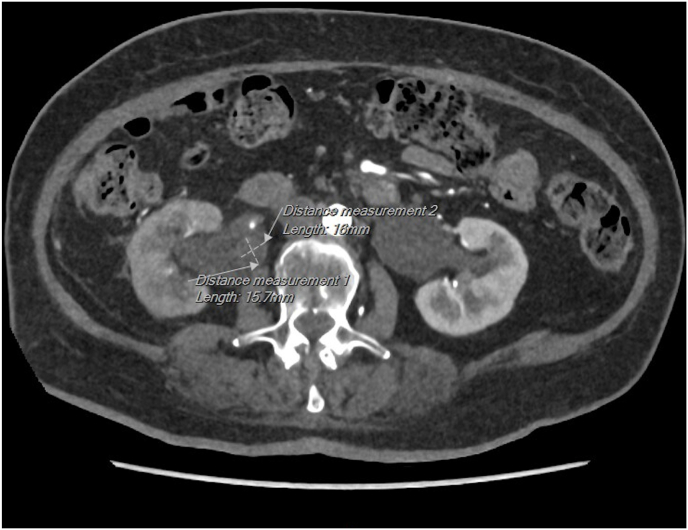
Axial (transverse) CT image in arterial phase showing a small obstructing calculus at the right ureteropelvic junction (UPJ), with associated dilation of the renal pelvis and calyces. The stone is measured in two dimensions.

**Fig. 2:**
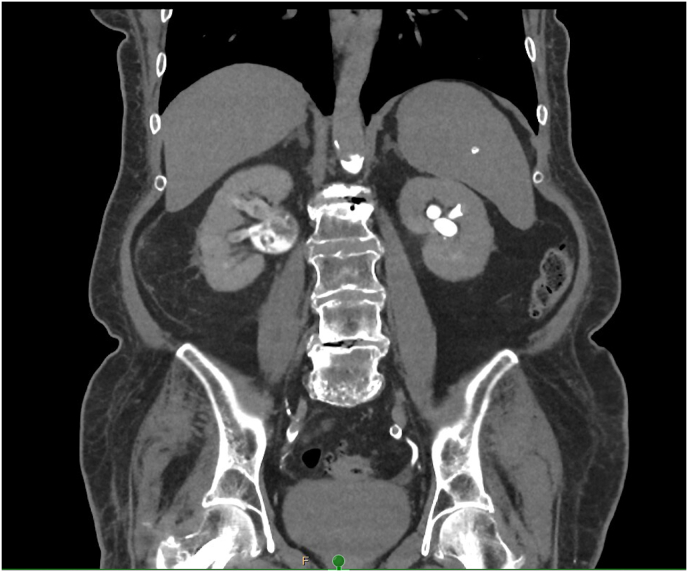
Coronal delayed-phase CT image (30 minutes post-contrast) demonstrating pooling of contrast in the right pyelocaliceal system without contrast passage into the ureter, consistent with complete obstruction at the UPJ.

**Fig. 3:**
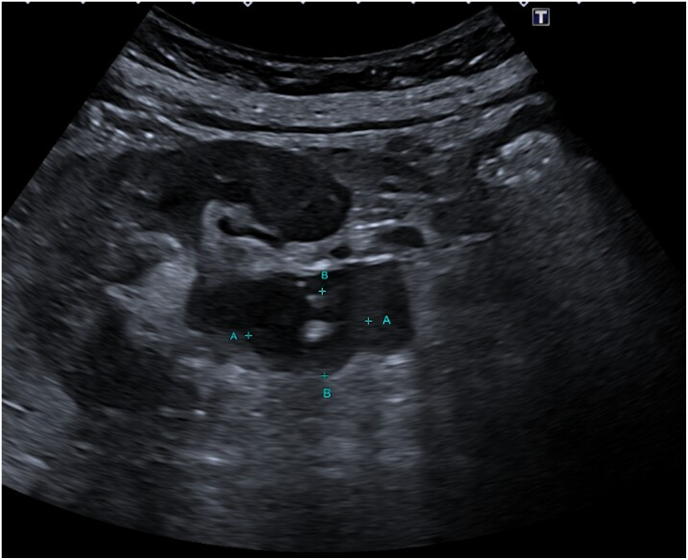
Ultrasound image of the right kidney showing a centrally located echogenic mass within the renal pelvis, surrounded by debris and without posterior acoustic shadowing or twinkle artifact. These findings are atypical for a calculus and are suggestive of a fungal ball.

Upon evaluation, she was hemodynamically stable with no signs of sepsis. Laboratory tests revealed leucocytosis and an elevated CRP. A CT scan of the abdomen was performed, including non-contrast, arterial, portal venous, and delayed phases. It confirmed an obstructing stone at the right ureteropelvic junction (UPJ) with no ureteral contrast passage on delayed imaging, suggesting complete obstruction (Fig. 1, Fig. 2). A subsequent ultrasound showed right-sided hydronephrosis and a centrally located echogenic mass in the renal pelvis, suspicious for a fungal ball (Fig. 3).

Given her acute kidney injury and obstructive findings, she was admitted to the inpatient ward. A double-J stent was placed, but symptoms persisted. Blood and urine cultures showed *Candida albicans*, prompting initiation of systemic antifungal therapy with fluconazole and caspofungin in consultation with a medical microbiologist.

Repeated ultrasound and CT imaging suggested an obstructing mass consistent with a fungal ball in the right renal pelvis. Given the patient's candidemia and risk of endovascular fungal seeding, further imaging was performed to exclude involvement of prosthetic material.

### 2.1 Procedure

After clinical stabilization, endoscopic intervention was performed using ureterorenoscopy. A bendable ureteral access sheath with an integrated suction device was introduced to facilitate irrigation and maintain low intrarenal pressure. Multiple fungal balls, with a soft, and adherent aspect, were visualized in the renal pelvis and calyces.

Initial attempts to remove these lesions using a traditional stone basket (Sacred Heart Medical HALO nitinol 1.5F basket) were unsuccessful, as the device fragmented the fungal balls without achieving en bloc extraction. In contrast, the suction device enabled intact removal of the fungal balls, significantly improving clearance efficiency and visibility. This technique proved especially valuable in this case and represents the core innovation of our approach.

A central stone was identified, serving as the nidus around which the fungal material had aggregated. Laser lithotripsy in dusting mode was used to disintegrate the central stone, with residual debris removed via suction. The procedure was completed without complications. Given the high fungal burden and friable debris, a temporary 7 Fr nephrostomy was placed at the end of the procedure to ensure dependable low-pressure upper-tract drainage and to enable targeted postoperative antifungal irrigation. This postoperative drainage strategy was agreed upon in multidisciplinary consultation (urology, nephrology, infectious diseases, medical microbiology and hospital pharmacy).

### 2.2 Outcome and follow-up

Postoperatively, the patient showed rapid clinical improvement. Blood and urine cultures cleared within days, and inflammatory markers normalized. Follow-up ultrasonography showed resolution of the renal obstruction. The nephrostomy and double-J stent were removed after a course of local and systemic antifungal therapy. No recurrence was observed during clinical follow-up.

## 3 Discussion

Fungal balls of the upper urinary tract pose unique therapeutic challenges. While systemic antifungal therapy is essential, it often fails to penetrate dense fungal masses or biofilms.^7^ Drainage alone may be insufficient, and ureteral stents can potentially exacerbate the obstruction. Surgical removal, though effective, can be technically demanding, especially in the context of limited visibility, friable tissue and mobile debris.

This case introduces a novel modification to standard endoscopic techniques: the use of a bendable ureteral access sheath with an integrated suction device during URS. The key innovation lies in the ability to remove fungal balls in toto, which significantly improved clearance efficiency compared to traditional basket retrieval. In this patient, initial attempts with a stone basket were unsuccessful due to fragmentation of the fungal material. The suction device enabled in toto extraction, reducing the risk of residual debris and the need for repeat procedures.

Importantly, this approach allowed for complete clearance of the collecting system without the need for percutaneous access or tract dilatation, as would be required in percutaneous nephrolithotomy (PNL). In our institution, although the number of cases is limited, PNL has generally been the preferred approach for fungal ball removal. However, in this case, the minimally invasive suction-assisted URS technique proved especially valuable in a high-risk, frail patient with candidemia and dual antiplatelet therapy, where more invasive options such as PNL posed significant bleeding risks. To our knowledge, this is the first documented case demonstrating in toto removal of renal fungal balls using suction-assisted URS. This technique represents a promising advancement in minimally invasive endourology, particularly for high-risk patients where conventional approaches such as PNL are contraindicated. By combining efficient irrigation, pressure control, and targeted removal, this approach may offer a safer and effective alternative in similarly complex cases. It should be mentioned that in this case the bleeding risk of placement of a 7 French PCN at the end of the procedure was accepted, to facilitate irrigation postoperatively.

## 4 Conclusion

We report a novel, minimally invasive technique for the endoscopic removal of renal fungal balls using a suction-enabled ureteral access sheath. This approach enabled complete removal of fungal material and efficient clearance of a concomitant central stone, minimizing the risk of residual debris and prolonged infection. Despite being a single case, this technique may prove beneficial in complex patients, particularly when conventional stenting and/or URS are insufficient and more invasive procedures such as percutaneous approach are relatively contraindicated.

## CRediT authorship contribution statement

**Mies van den Biggelaar:** Writing – review & editing, Writing – original draft, Data curation. **Manon te Dorsthorst:** Writing – review & editing, Conceptualization. **Xiaoye Zhu:** Writing – review & editing, Conceptualization. **Frank d'Ancona:** Writing – review & editing, Conceptualization.

## Funding statement

No funding was received for this study.

## Conflict of interest statement

The authors declare no conflicts of interest.
